# A clinical challenging situation of Intra oral fibroma mimicking pyogenic granuloma

**DOI:** 10.11604/pamj.2015.22.263.8080

**Published:** 2015-11-19

**Authors:** Velavan Krishnan, Karthik Shunmugavelu

**Affiliations:** 1 Consultant Oral and Maxillofacial Surgeon, Saraswathy Multispeciality Hospital, Balaiah Garden, Madipakkam, Chennai, Tamil Nadu 600091, India; 2 Consultant Dental Surgeon, Saraswathy Multispeciality Hospital, Balaiah Garden, Madipakkam, Chennai, Tamil Nadu 600091, India

**Keywords:** Fibroma mimicking Pyogenic Granuloma, orthopantomograph, maxillary occlusal

## Image in medicine

A 45 year old female presented to the Department of Dentistry and Faciomaxillary Surgery complaining of difficulty in swallowing and tongue movements. General health examination revealed that the patient was conscious, oriented, a febrile and vitals were stable. Occlusion stable. Mouth opening was of three finger breadth. Lateral temporomandibular joint movements were satisfactory. On intra oral clinical examination, a pedunculated mass was observed palatally in relation to left maxillary molar teeth region, where a root stump was evident. Patient stated that the swelling has started as a pinpoint lesion before 1 year and progressed to the size of 5cm x 3cm as seen during clinical examination. The colour of the mass was of coral pink, smooth, nodular in some areas and symptomatic during contact between maxillary and mandibular teeth. Radiological investigations such as orthopantomograph and maxillary occlusal views revealed absence of bony involvement. To differentiate the clinical mimicking situation of intra oral fibroma and pyogenic granuloma, surgical excision was done followed by histopathological examination reporting as intra oral fibroma. Fibroma is of smooth surfaced painless solid mass, occasionally nodular, with collagen and connective tissue histopathologically. Whereas, pyogenic granuloma present as painless and smooth mass which bleeds on palpation clinically with histopathological picture of chronic inflammatory infiltrate, endothelial cell proliferation and hyperplastic granulation tissue. Postoperative review was after 1 month with good prognosis thereby facilitating efficient swallowing and tongue movements.

**Figure 1:**
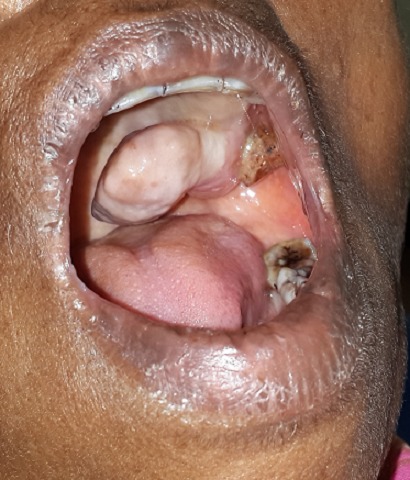
Clinical view of intra oral mass in relation of left maxillary molar region

